# Reference values for low muscle mass and myosteatosis using tomographic muscle measurements in living kidney donors

**DOI:** 10.1038/s41598-023-33041-1

**Published:** 2023-04-10

**Authors:** Lisa B. Westenberg, Marcel Zorgdrager, Tim D. A. Swaab, Marco van Londen, Stephan J. L. Bakker, Henri G. D. Leuvenink, Alain R. Viddeleer, Robert A. Pol

**Affiliations:** 1 Division of Transplant Surgery, Department of Surgery, University Medical Centre Groningen, University of Groningen, PO Box 30 001, 9700 RB Groningen, The Netherlands; 2 Department of Radiology, Medical Imaging Center, University Medical Centre Groningen, University of Groningen, Groningen, The Netherlands; 3 Division of Nephrology, Department of Internal Medicine, University Medical Centre Groningen, University of Groningen, Groningen, The Netherlands

**Keywords:** Skeletal muscle, Computed tomography, Kidney

## Abstract

Low muscle mass and myosteatosis are associated with poor clinical outcomes. Computed tomography (CT) imaging is an objective method for muscle mass and quality assessment; however consensus on cut-off values is lacking. This study assessed age-, sex-, and body mass index (BMI)-specific reference values of skeletal muscle parameters and correlated muscle mass with 24-h urinary creatinine excretion (24-h UCE). In total, 960 healthy subjects were included in this study. Muscle mass and quality were determined using axial CT slices at the vertebral level L3. The muscle area was indexed for height (skeletal muscle index [SMI]). The mean age was 53 ± 11 years, and 50% were male. The SMI reference values for low muscle mass in males were 38.8 cm^2^/m^2^ (20–29 years), 39.2 (30–39 years), 39.9 (40–49 years), 39.0 (50–59 years), 37.0 (60–69 years), and 36.8 (70–79 years). For females, these reference values were 37.5 cm^2^/m^2^ (20–29 years), 35.5 (30–39 years), 32.8 (40–49 years), 33.2 (50–59 years), 31.2 (60–69 years), and 31.5 (70–79 years). 24-h UCE and SMI were significantly correlated (*r* = 0.54, *p* < 0.001) without bias between the two methods of assessing muscle mass. This study provides age-, sex-, and BMI-specific reference values for skeletal muscle parameters that will support clinical decision making.

## Introduction

Low skeletal muscle mass is an important risk factor for mortality and morbidity in older people^1^ and multiple other populations^2–4^. Fat infiltration of skeletal muscle, also known as myosteatosis, has also been associated with poor clinical outcomes^5^. Determining the presence of low muscle mass and myosteatosis may aid in assessing whether a patient is fit for invasive treatments, surgery, and medication, with their potential side effects^1,6^.

The best methods to adequately assess muscle mass, quality, and the presence of low muscle mass and myosteatosis have been a topic of much debate^7^ in the past decade. Different strategies to determine muscle mass have been proposed, of which bioelectrical impedance analysis, dual-energy X-ray absorptiometry (DEXA), and cross-sectional computed tomography (CT) are most commonly used^7–9^. 24-h urinary creatine excretion rate (24-h UCE) is another well-known marker of muscle mass^10^. Although CT imaging can only be used opportunistically due to radiation exposure, this technique has proven to be an accurate and objective way for muscle mass and quality assessment and with advancements in artificial intelligence its accuracy and speed are rapidly increasing. Previous studies have proposed cut-off values for low muscle mass using cross-sectional CT imaging at vertebral level L3 in healthy subjects of different ethnicities^11–15^. For these cut-off values, the lumbar skeletal muscle area (SMA) and height-corrected skeletal muscle index (SMI) are the most widely implemented, and low SMI is used as a marker for low muscle mass. Low mean skeletal muscle radiation attenuation (SMRA), or muscle density, can be used as a marker for myosteatosis. Because muscle mass is dependent on age, sex, and BMI, large variations exist in cut-offs^11–15^. The cut-off values determined in various populations are often not corrected for these variables, which could result in over- or underestimation of the presence of low muscle mass and its associated risk factors. Subsequently, patients with sarcopenia who are not identified as such may undergo (surgical) treatment where safer alternatives should be sought, and patients incorrectly identified as sarcopenic may be denied important treatment options. The purpose of this study was to assess age-, sex-, and BMI-specific reference values for skeletal muscle parameters in a large healthy Caucasian population, and to correlate SMI with 24-h UCE.

## Methods

In this retrospective cohort study, 960 healthy subjects were recruited from potential living kidney donors at the University Medical Center Groningen (UMCG) between 2002 and 2019. Living kidney donors need to be relatively healthy (e.g., absence of manifested diabetes mellitus, major cardiovascular risk factors, recent or active malignancies, chronic/active infection, hypertension with end-organ damage, or inadequately regulated hypertension) and undergo a thorough screening process (including CT imaging) to be able to donate; therefore, living kidney donors provide the opportunity to identify reference values for low muscle mass and quality. Subjects were excluded if they were unable to provide informed consent, significant interfering artefacts on CT imaging were present and/or when the abdominal wall muscles were not fully visualized. All clinical, biochemical, and radiological data were collected as part of the TransplantLines Biobank and Cohort Study (ClinicalTrials.gov identifier: NCT03272841)^16^. All participants provided written informed consent for enrolment. The TransplantLines study protocol was approved by the local Institutional Ethical Review Board (‘Medisch Ethische Toetsingscommissie UMC Groningen’, METc 2014/077), adheres to the UMCG Biobank Regulation, and is in accordance with the WMA Declaration of Helsinki and the Declaration of Istanbul^16^. In 2020, historical data of transplantation patients and donors were included in the TransplantLines biobank and cohort study and underwent a renewed ethical review in accordance with the current ethical guidelines. With its approval, the use of historical clinical and biological materials of transplant recipients and donors in research and publications was approved alongside newly collected data and samples of transplant recipients and donors. The application for access to the necessary retrospective data for this study was approved by the TransplantLines working group, as the analyses in the present study fall under the scope of the METc. The estimated glomerular filtration rate (eGFR, mL/min/1.73 m^2^) was calculated following the CKD-EPI equation^17^, and the measured glomerular filtration rate (mGFR, mL/min) was calculated from measurements of the clearance of radiolabeled iothalamate (^125^I-iothoalamate) as described previously^18^. Body composition measurements consisted of body surface area (BSA, m^2^), calculated using the Du Bois and Du Bois formula^19^, and BMI (kg/m^2^). All clinical and biochemical measurements were performed as previously described^16^.

All subjects underwent CT imaging at the UMCG (n = 932) or non-academic referral hospitals (n = 28) in the Netherlands. All scans were contrast-enhanced (portal venous phase, n = 12; arterial phase, n = 38; late phase, n = 910). The slice thickness varied between 0.75 and 5 mm. The tube voltage and current varied between 70 and 150 kVp (median 100 kVp, IQR 100 kVp) and 20–455 mAs (mean 98 mAs, SD 48 mAs), respectively.

**Figure 1:**
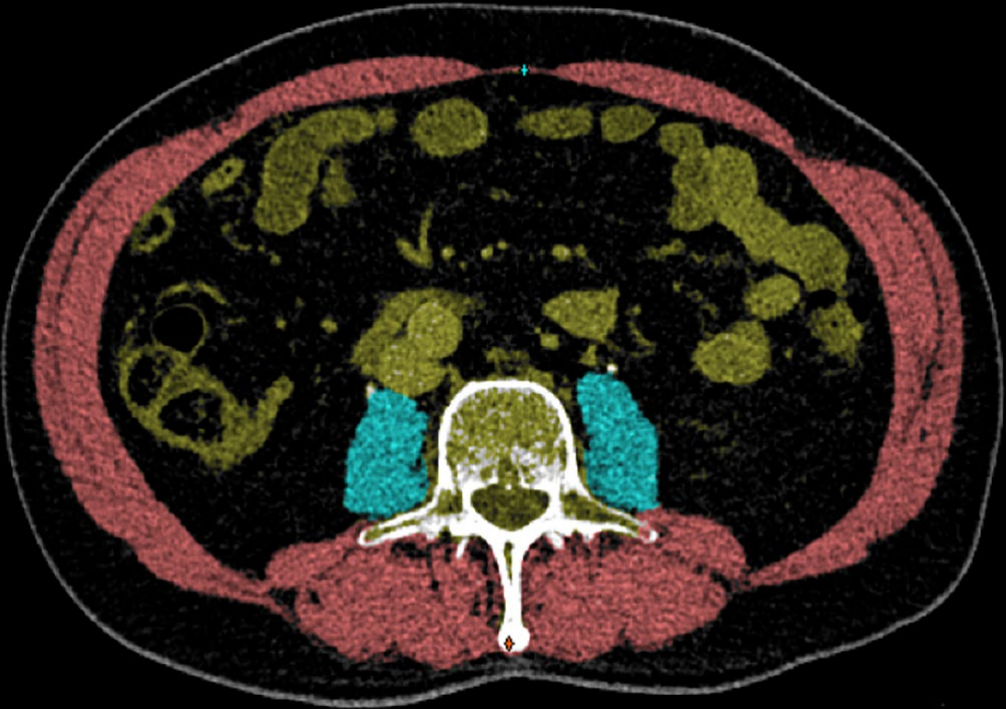
Cross-sectional CT image in the semi-automatic program SarcoMeas. Illustrating the artificial intelligence stratification of abdominal wall and paraspinal muscles (red), left and right psoas muscles (blue), and abdominal viscera (yellow), based on radiographic density in Hounsfield Units.

The cross-sectional area of the skeletal muscle was determined at level L3 and included the psoas, paraspinal, and abdominal wall muscles. The cross-sectional plane was analysed using a semi-automatic program (Sarcomeas, version 0.54, UMCG, Groningen) in which slices were imported anonymously from the picture archiving and communication system (PACS) in the native Digital Imaging and Communications in Medicine (DICOM) format (Fig. 1). Two experienced radiologists (MZ and AV) manually delineated the muscle area on vertebral level L3 at the slice where both transverse processes were visible. Within these outlines, the muscle was defined by selecting voxels with densities ranging from -29 to + 150 Hounsfield units (HU). The skeletal muscle area (SMA, cm^2^) was indexed for height (SMI, cm^2^/m^2^), which was considered an indicator of muscle mass. The mean SMRA was defined as the mean HU of all pixels of the total SMA, which was considered an indicator of muscle quality. Measurements were performed for each muscle compartment and for the total muscle area.

Continuous variables with normal distribution are presented as mean (standard deviation) and analysed using Student’s t-test. Reference values for low muscle mass were determined by the mean SMI value minus two standard deviations, as well as an SMI value below the 5^th^ percentile (*p5*), which are the standard methods for defining the cut-off^20^. These reference values are presented for age, sex, and BMI categories. BMI was stratified according to the current World Health Organization guidelines^21^. Eight subjects had a BMI < 18.5 kg/m^2^ and six subjects had a BMI ≥ 35 kg/m^2^. Therefore, we report the values for three BMI categories (< 25, 25–29.99, and ≥ 30 kg/m^2^). Low muscle density, a marker of myosteatosis, was determined using SMRA. For the determination of SMRA and its reference values, we excluded all four cases with an unenhanced CT scan because the use of contrast agents can affect SMRA (but not SMA or SMI)^22^. This involved four female participants (two in the category 50–59 years and two in the category 60–69 years). Correlation analyses were performed using Pearson product-moment correlation coefficient, and agreement between CT analysis and biochemical analysis of muscle mass (i.e., 24-h UCE) was investigated using Bland–Altman plots in which mean differences in Z-scores were analysed (95% confidence interval, CI). 24-h urinary creatinine excretion data were collected within 1 week before or after cross-sectional imaging. Two-tailed values were used, and the significance level was set at p < 0.05. Statistical analyses were performed using SPSS® version 27 (IBM, Armonk, NY, USA). Missing data were limited in this study (< 5%). The variables included in our analyses with missing data were BMI and 24-h UCE. Only four out of 960 subjects had missing BMI data. Multiple imputation following the fully conditional specification method for 24-h UCE did not change our results.

## Results

### Study population

A total of 960 healthy subjects were included in this study, of which 50% were male. Donors were stratified by age (i.e., 20–29, 30–39, 40–49, 50–59, 60–69, 70–79 years). Males had significantly higher values for all baseline characteristics except BMI, use of antihypertensives, and history of diabetes (Table 1). Male subjects also had significantly higher mean SMA, SMI, and SMRA values than female subjects (Supplementary Table S1). Mean SMA showed a negative linear correlation with age in both males and females (males: *r* = −0.26, *p* < 0.001; females: *r* = −0.39, *p* < 0.001) (Supplementary Fig. S1A,B), and the mean SMI was lower with increasing age (males: *r* = −0.13, *p* = 0.004; females: *r* = −0.23, *p* < 0.001) (Supplementary Fig. S1C,D). SMRA showed a negative linear correlation with age in both sexes (males: *r* = −0.38, *p* < 0.001; females: *r* = −0.38, *p* < 0.001) (Supplementary Fig. S1E,F). Univariable linear regression analyses with age and the different skeletal muscle parameters showed significant negative associations between age and SMA (males: Β = −0.53, p < 0.001; females: Β = −0.53, p < 0.001), SMI (males: Β = −0.08, p = 0.004; females: Β = −0.11, p < 0.001), and SMRA (males: Β = −0.24, p < 0.001, females: Β = −0.30; p < 0.001) in both male and female subjects (Table 2).

**Table 1:** Baseline characteristics of the study population.

	Total population		*p*
	Male (n = 478)	Female (n = 482)	
Age, years	52 ± 12	54 ± 10	0.01
Weight, kg	87.0 ± 11.7	73.8 ± 10.9	< 0.001
Height, cm	182.0 ± 7.21	168.7 ± 6.6	< 0.001
BMI, kg/m^2^	26.3 ± 3.3	25.9 ± 3.6	0.13
BSA, m^2^	2.1 ± 0.2	1.8 ± 0.1	< 0.001
SBP, mmHg	129.5 ± 12.9	123.6 ± 12.3	< 0.001
DBP, mmHg	77.9 ± 9.14	73.6 ± 8.72	< 0.001
Hypertension^a^, n (%)	95 (20.0)	56 (11.7)	< 0.001
Use of antihypertensives, n (%)	79 (16.7)	64 (13.4)	0.16
Fasting glucose, mmol/L	5.46 ± 0.64	5.28 ± 0.52	< 0.001
History of diabetes, n (%)	6 (1.3)	5 (1.0)	0.76
Metabolic syndrome^b^, n (%)	91 (27.2)	52 (15.7)	< 0.001
24 h UCE, mmol/24 h	16.2 ± 3.9	11.0 ± 3.6	< 0.001
Serum creatinine, µmol/L	84.7 ± 12.2	67.9 ± 9.1	< 0.001
eGFR, mL/min/1.73 m^2^	89.6 ± 15.7	85.0 ± 14.4	< 0.001
mGFR, mL/min	121.7 ± 20.0	103.7 ± 17.8	< 0.001

Values of variables are given as mean ± standard deviation or number (percentage).

Statistical significance is displayed for male vs female.

*BMI* body mass index (kg/m^2^), *BSA* body surface area (m^2^), *SBP* systolic blood pressure (mmHg), *DBP* diastolic blood pressure (mmHg), *UCE* urinary creatinine excretion (mmol/24 h), *eGFR* estimated glomerular filtration rate (mL/min/1.73m^2^), *mGFR* measured Glomerular Filtration Rate (mL/min × 1.73m^2^).

^a^SBP >140 mmHg and/or DBP >90 mmHg.

^b^According to the modified criteria of the National Cholesterol Education Program Adult Treatment Panel III (NCEP/ATPIII).

**Table 2:** Univariable linear regression analyses of age and 24-UCE with skeletal muscle parameters.

	SMI, cm^2^/m^2^				SMA, cm^2^				SMRA, HU			
	B (95% CI)	Std. β	p-value	Adj. R^2^	B (95% CI)	Std. β	p-value	Adj. R^2^	B (95% CI)	Std. β	p-value	Adj. R^2^
Age, years												
Males (n = 478)	−0.08 (−0.14 to −0.03)	−0.13	0.004	0.02	−0.53 (−0.70 to −0.35)	−0.26	< 0.001	0.07	−0.24 (−0.30 to −0.19)	−0.38	< 0.001	0.14
Females (n = 482)	−0.11 (−0.15 to −0.07)	−0.23	< 0.001	0.05	−0.53 (−0.64 to −0.42)	−0.39	< 0.001	0.15	−0.30 (−0.36 to −0.23)	−0.38	< 0.001	0.15
24-h UCE, mmol/24 h	0.97 (0.87–1.07)	0.54	< 0.001	0.29	4.86 (4.49–5.23)	0.65	< 0.001	0.43	0.10 (−0.01 to 0.21)	0.06	0.08	0.002

Univariable linear regression analyses of age with skeletal muscle parameters were performed for male and female subjects separately.

*SMI* skeletal muscle index (cm^2^/m^2^), *SMA* skeletal muscle area (cm^2^), *SMRA* skeletal muscle radiation attenuation (HU), *24-h* *UCE* 24-h urinary creatinine excretion (mmol/24 h).

### Reference values for low muscle mass and muscle density

The reference values (calculated as mean minus two standard deviations) for low muscle mass (SMI) in males were 38.8 cm^2^/m^2^ (20–29 years), 39.2 (30–39 years), 39.9 (40–49 years), 39.0 (50–59 years), 37.0 (60–69 years), and 36.8 (70–79 years) (Table 3). For females, reference values of SMI were 37.5 cm^2^/m^2^ (20–29 years), 35.5 (30–39 years), 32.8 (40–49 years), 33.2 (50–59 years), 31.2 (60–69 years), and 31.5 (70–79 years). The *p5* reference values are presented in Table 4. The reference values (mean − 2SD) for low muscle density (SMRA) in males were 45.0 HU (20–29 years), 39.6 (30–39 years), 37.0 (40–49 years), 34.9 (50–59 years), 32.6 (60–69 years), 29.7 (70–79 years) (Table 3). For females, SMRA reference values were 32.5 HU (20–29 years), 38.4 (30–39 years), 38.1 (40–49 years), 32.6 (50–59 years), 27.7 (60–69 years), 31.8 (70–79 years).

**Table 3:** Reference values (mean-2SD) for SMI, SMA, and SMRA, per age and BMI category.

Age, years	20–29		30–39		40–49		50–59		60–69		70–79	
	Male	Female	Male	Female	Male	Female	Male	Female	Male	Female	Male	Female
SMI, cm^2^/m^2^												
All BMI’s	38.8	37.5	39.2	35.5	39.9	32.8	39.0	33.2	37.0	31.2	36.8	31.5
< 25	38.3	–^a^	37.7	34.9	39.0	32.6	37.7	32.6	35.0	31.4	31.1	32.7
25–29.99	43.0	–^a^	44.4	38.4	43.1	33.1	41.0	34.2	40.6	31.4	39.8	29.0
≥ 30	38.9	–^a^	42.9	36.6	44.3	38.1	46.5	34.5	45.3	31.9	48.5	39.8
SMA, cm^2^												
All BMI’s	130.6	103.0	140.5	101.3	133.7	98.3	132.1	94.4	121.8	88.7	115.5	84.8
< 25	125.1	–^a^	134.5	103.8	137.8	95.6	123.3	93.4	113.4	87.7	101.0	85.5
25–29.99	142.5	–^a^	156.6	105.3	136.8	99.8	141.9	95.5	134.0	89.7	124.3	82.6
≥ 30	132.6	–^a^	155.1	89.3	136.8	114.4	143.6	95.2	137.9	92.4	135.1	86.1
SMRA, HU												
All BMI’s	45.0	32.5	39.6	38.4	37.0	38.1	34.9	32.6	32.6	27.7	29.7	31.8
< 25	48.8	–^a^	46.0	42.8	41.2	41.7	42.1	41.1	37.7	35.1	39.3	34.8
25–29.99	46.2	–^a^	39.4	42.0	39.7	41.1	33.9	32.0	31.4	27.3	32.3	30.6
≥ 30	41.1	–^a^	31.0	32.6	30.6	28.5	32.1	27.1	35.4	21.1	22.9	33.4

*SMI* skeletal muscle index (cm^2^/m^2^), *SMA* skeletal muscle area (cm^2^), *SMRA* skeletal muscle radiation attenuation (HU), *BMI* body mass index (kg/m^2^).

^a^Number of donors per BMI category too small to perform analyses. Only reference value for all BMI’s provided.

**Table 4:** Reference values (*p5*) for SMI, SMA, and SMRA, per age and BMI category.

Age, years	20–29		30–39		40–49		50–59		60–69		70–79	
	Male	Female	Male	Female	Male	Female	Male	Female	Male	Female	Male	Female
SMI, cm^2^/m^2^												
All BMI’s	43.1	40.7	41.8	36.9	43.1	35.4	41.3	35.4	40.5	34.1	33.3	31.5
< 25	43.1	–^a^	38.3	36.8	38.2	35.1	39.8	34.1	40.0	34.2	30.9	31.6
25–29.99	50.0	–^a^	47.6	37.9	43.5	35.7	44.9	35.8	43.8	33.5	43.5	31.5
≥ 30	46.9	–^a^	46.4	40.0	44.9	39.4	46.4	36.7	52.5	35.5	51.0	42.7
SMA, cm^2^												
All BMI’s	129.5	110.8	143.8	107.4	141.6	102.0	136.5	98.5	134.8	94.7	109.8	86.3
< 25	129.5	–^a^	141.0	104.1	140.4	100.0	129.1	97.5	126.8	94.4	101.3	82.9
25–29.99	167.6	–^a^	164.7	108.3	134.2	98.8	147.1	99.4	140.0	94.2	133.1	96.4
≥ 30	155.3	–^a^	169.3	111.6	139.1	112.2	136.7	98.9	162.1	97.3	148.1	100.0
SMRA, HU												
All BMI’s	47.3	42.3	42.2	36.3	38.3	40.7	36.9	33.2	32.5	31.7	32.1	34.8
< 25	49.2	–^a^	45.6	45.1	45.0	42.6	44.6	43.7	38.4	38.0	39.1	38.2
25–29.99	52.7	–^a^	42.1	43.7	41.1	41.4	36.6	33.1	31.4	30.1	35.9	34.6
≥ 30	47.3	–^a^	36.7	36.8	34.8	24.8	29.6	26.2	36.5	21.2	29.7	37.3

*SMI* skeletal muscle index (cm^2^/m^2^), *SMA* skeletal muscle area (cm^2^), *SMRA* skeletal muscle radiation attenuation (HU), *BMI* body mass index (kg/m^2^).

^a^Number of donors per BMI category too small to perform analyses. Only reference value for all BMI’s provided.

**Figure 2:**
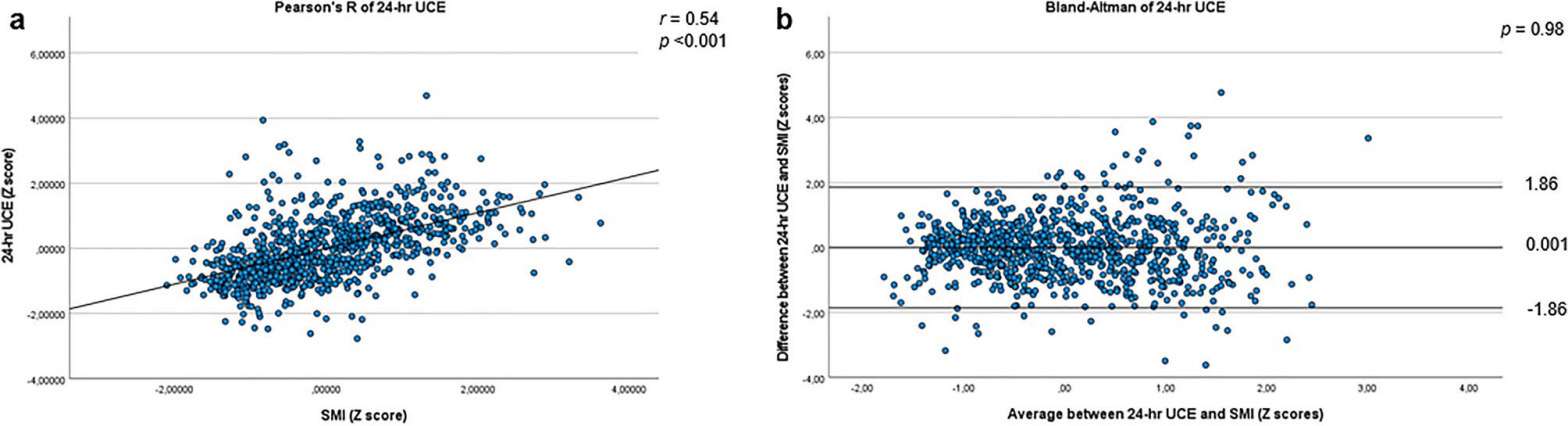
Pearson's correlation and Bland–Altman plots of 24-h UCE and SMI. (**A**) Pearson’s correlation of 24 h urinary creatinine excretion (24-h UCE) and skeletal muscle index (SMI); (**B**) Bland–Altman plot of 24 h UCE and SMI. The middle horizontal line in the Bland–Altman plot shows the mean of the differences (= bias) between the two methods, and the outer two horizontal lines show the upper and lower 95% limits of agreement (= bias ± 1.96 × SD). *r*: Pearson’s r.

In univariable linear regression analyses, 24-h UCE was significantly associated with measures of skeletal muscle quantity (SMA and SMI), but not with SMRA, the measure for skeletal muscle quality (Table 2). 24-h UCE and SMI were significantly correlated (*r* = 0.54, *p* < 0.001) with high levels of SMI being correlated with higher levels of 24-h UCE (Fig. 2A). A Bland–Altman plot was used to test the agreement between SMI and 24-h UCE. There was no significant mean difference between the two methods for assessing muscle mass (mean = 0.001, SD = 0.95, *p* = 0.98) (Fig. 2B). BMI was positively correlated with SMI (*r* = 0.35, *p* < 0.001; Supplementary Fig. S2A). For BMI, Bland–Altman plots also showed no significant mean difference compared to SMI (mean = −0.005, SD = 1.13, *p* = 0.89) (Supplementary Fig. S2B).

## Discussion

In this study, we provide age-, sex-, and BMI-specific reference values for skeletal muscle parameters in a large healthy Caucasian population and correlate SMI with 24-h UCE. A consensus on cut-off values for (low) muscle mass and density is currently lacking, impairing the clinical recognition of patients at risk of low muscle mass and myosteatosis, for which reference values are required to determine what is aberrant or pathological. The population is changing, with older individuals requiring optimization of medical assessment and personalized strategies to preserve good treatment outcomes.

With the exception of BMI, male subjects showed significantly higher anthropometric parameters than did female subjects. Male participants also showed higher 24-h UCE and eGFR values. Frequently used reference values for 24-h UCE and mGFR, and more recently proposed sex-specific reference values, also reported higher values for males than females^23,24^. It is widely known that body composition differs between males and females, with men generally having more muscle mass^25^. In our study, the mean SMA and SMI were significantly higher in male subjects than in female subjects across all age categories. This is in line with previous studies performed in healthy Caucasian and Asian populations^12,13,15,16^. The SMI in healthy Caucasian males was 1.31-fold higher than that in females^13^. In a healthy Asian study population, the psoas muscle mass index was 1.53-fold higher in males than in females^16^. In our study population, SMI in males was 1.26-fold higher than that in females.

Older age was significantly correlated and associated with lower SMA and SMI values in this study. Skeletal muscle mass decreases with advancing age owing to a decrease in muscle protein synthesis^26^ and loss of type II fibres^27^. It has previously been suggested that this loss of skeletal muscle mass is most prevalent in individuals ≥ 70 years old, with a 0.5–1.0% loss of skeletal muscle mass per year after the age of 70^28^, and a 15% decrease in the cross-sectional area of the thigh over a 12-year period starting at 65.4 ± 4.2 years of age^29^. Comparable results have been reported previously in which a 1.20-fold higher SMI was found in Asian individuals aged < 50 years vs. ≥ 50 years^16^. In a Caucasian study population, SMI was 1.08-fold higher in individuals aged < 50 years than in those aged ≥ 50 years^13^. In our study population, SMI was 1.06-fold higher in subjects aged < 50 years than in those aged ≥ 50 years.

SMRA, expressed in Hounsfield Units, can be reliably determined using CT analysis. Lower values reflect increased muscle lipid content, which is observed in people with comorbidities, such as obesity, type 2 diabetes, and cancer^30^. Low muscle density also reflects the presence of myosteatosis, which is associated with adverse postoperative outcomes^7^. A study by Anderson et al. showed that muscle attenuation was generally lower in females than in males, with variations between muscle groups^31^. Although our study measured different skeletal muscles, our findings also showed lower SMRA in females than in males. In addition, older age was significantly correlated and associated with lower SMRA in both males and females in our cohort. This is in line with other studies investigating SMRA in relation to age, in which older adults (75–87 years) had significantly lower muscle attenuation in all muscles^31^.

Few studies have described reference values for skeletal muscle mass measured using CT analysis at the L3 vertebral level in a healthy population. Of these studies, only a small proportion reported reference values for SMA, SMI, and SMRA stratified by age, which is of clinical significance given the natural decline in muscle mass and function with age. When comparing reported reference values for (low) muscle mass, it becomes apparent that some important differences exist in the methods of reporting, from two standard deviations below the mean to reporting *p5* values. To facilitate juxtaposition, we reported the reference values using both methods. Van der Werf et al.^13^ and van Vugt et al.^32^ provide predicted *p5* values for L3 level SMA, SMI, and SMRA per age category that slightly differ from our results. Ufuk et al. described *p5* values for L3 level SMA and SMI in healthy adults aged 20–40 years and 20–60 years^14^. The range of SMA and SMI *p5* values is similar to our findings, although it is difficult to compare these results because the age categories were not identical. The latter also applies to the findings of Derstine et al., who provided reference values that are two standard deviations below the mean for SMA, SMI, and SMRA at the L3 level for ages 18–40 years^12^. Differences could be explained by variations in patient characteristics, differences in scanners and image acquisition parameters, type and amount of contrast, contrast timing, and amount of experience in manually delineating the muscle area on the vertebral level L3 slice.

The positive correlation between SMI and BMI found in our study is in line with the finding that individuals with higher muscle mass tend to have a higher BMI. The use of BMI in assessing health risk is encountering increasing scrutiny since this anthropometric measure does not adequately reflect differences in body composition. Therefore, the health risk of obese individuals with high muscle mass might be overestimated, whereas the health risk of obese individuals with low muscle mass (also known as sarcopenic obesity) might be underestimated^33^. This contradiction may also apply to individuals of normal weight, for whom radiological analysis using CT scans can be superior to BMI in assessing the actual body composition and potential imbalance with the associated health risks. Radiological analysis of body composition using CT scans may further aid treatment decisions for patients undergoing surgery. For many (surgical) indications, CT scans are performed routinely, resulting in no additional radiation exposure while adding valuable information aiding in individualized surgical decision making and allowing for prehabilitation.

To address the possible influence of BMI on skeletal muscle parameters, our study also reported BMI-specific values for SMA, SMI, and SMRA. Recently, Derstine et al. proposed the use of BMI-adjusted z-scores of height-adjusted SMA values^34^, to distinguish between ‘more muscular’ and ‘less muscular’ body compositions at any BMI. Although it is an interesting and promising method, it requires further validation. In addition, van Vugt et al.^32^ proposed the use of nomograms incorporating BMI to calculate the estimated healthy skeletal muscle mass of individuals in patient populations. While another promising approach, most body weight and height measures were self-reported, and further validation is necessary. In this reference paper, we present our results stratified by age, sex, and BMI.

This study showed the presence of a strong positive correlation and association between 24-h UCE and SMI. The Bland–Altman plot showed agreement between SMI and 24-h UCE, with no significant mean difference between these two methods. This supports that CT analysis is an accurate method for radiological analysis of skeletal muscle mass in patients that require assessment of body composition^10,35^. However, if a CT scan is not available or desirable, 24-h UCE may be a reasonable alternative.

The strengths of this study are its large cohort with a wide spectrum of ages, lack of comorbidities, and standardized screening protocol. There are also a few limitations that need to be addressed, such as the retrospective study design, which could lead to less reliable results than a prospective design. In addition, there were differences in the number of subjects per age category, hindering the possibility of comparison between these categories. Only six subjects in this study had a BMI > 35 kg/m^2^, hampering generalizability to more obese individuals in which skeletal muscle mass measurements may be valuable. In addition to sex and age, SMRA may also be influenced by technical factors such as tube voltage (kVp), current (mAs), and the use, amount, and timing of the contrast agent and phase. Our reference values have not yet been analysed for clinical outcomes, which is necessary for translating these data to daily clinical practice. Our group is currently analysing our reference values against clinical outcomes in renal transplant recipients.

In conclusion, this study provides age-, sex-, and BMI-specific reference values for skeletal muscle parameters, and facilitates the interpretation of skeletal muscle mass and quality in healthy and diseased individuals. There was a strong positive correlation between CT-derived SMI values and 24-h UCE, with no significant mean difference between the two methods of assessing skeletal muscle mass. The results of this study can contribute to the comprehension of normal skeletal muscle mass and quality, and can act as reference data in clinical practice and future studies assessing the presence of low muscle mass and myosteatosis.

## Supplementary Information

Supplementary Information.

## Data Availability

Data described in the manuscript, code book, and analytic code will be made available upon request. Please contact dr. R.A. Pol, r.pol@umcg.nl.
