# Supplementary material for: Reference values for low muscle mass and myosteatosis using tomographic muscle measurements in living kidney donors
Source: Sci Rep. 2023 Apr 10;13:5835. doi: 10.1038/s41598-023-33041-1 (PMC10086018; doi:10.1038/s41598-023-33041-1)
Supplement: Supplementary file 1 — Supplementary Information. [file 41598_2023_33041_MOESM1_ESM.pdf]

Supplements

Manuscript title: Reference values for low muscle mass and myosteatosis using tomographic muscle measurements in living kidney donors

Authors: Lisa B. Westenberg#, Marcel Zorgdrager#, Tim D.A. Swaab, Marco van Londen, Stephan J.L. Bakker, Henri G.D. Leuvenink, Alain R. Viddeleer, Robert A. Pol

Table S1. CT muscle mass measurements, total and per age category.

Age, years	Total population			20-29		30-39		40-49		50-59		60-69		70-79	
	Male<br>n = 478	Female<br>n = 482	*p*	Male<br>n = 16	Female<br>n = 4	Male<br>n = 61	Female<br>n = 35	Male<br>n = 101	Female<br>n = 107	Male<br>n = 169	Female<br>n = 184	Male<br>n = 104	Female<br>n = 128	Male<br>n = 27	Female<br>n = 24
**Skeletal Muscle Area, cm^2**	175.4 ± 23.6	119.3 ± 13.7	<0.001	175.4 ± 22.4	126.2 ± 11.6	186.3 ± 22.9	128.9 ± 13.8	181.1 ± 23.7	124.3 ± 13.0	175.5 ± 21.7	119.8 ± 12.7	168.0 ± 23.1	113.1 ± 12.2	157.5 ± 21.0	111.0 ± 13.1
**Skeletal Muscle Index, cm^2/m^2**	53.1 ± 7.28	42.0 ± 4.75	<0.001	53.9 ± 7.53	43.4 ± 2.96	54.0 ± 7.40	44.0 ± 4.25	54.4 ± 7.26	43.0 ± 5.09	53.1 ± 7.07	42.1 ± 4.43	51.9 ± 7.44	40.4 ± 4.60	49.8 ± 6.48	41.7 ± 5.10
**Total abdominal wall muscle index, cm^2/m^2**	45.9 ± 6.36	37.0 ± 4.23	<0.001	45.8 ± 6.23	38.0 ± 2.89	46.2 ± 6.47	38.5 ± 3.63	46.9 ± 6.11	37.8 ± 4.55	46.1 ± 6.36	37.2 ± 4.00	45.0 ± 6.64	35.5 ± 4.09	43.4 ± 5.49	37.0 ± 4.37
**Right m. iliopsoas muscle index, cm^2/m^2**	3.51 ± 0.78	2.46 ± 0.54	<0.001	4.01 ± 1.08	2.69 ± 0.45	3.84 ± 0.78	2.74 ± 0.53	3.66 ± 0.81	2.55 ± 0.52	3.40 ± 0.69	2.40 ± 0.51	3.38 ± 0.74	2.39 ± 0.53	3.10 ± 0.72	2.33 ± 0.66
**Left m. iliopsoas muscle index, cm^2/m^2**	3.67 ± 0.79	2.56 ± 0.53	<0.001	4.09 ± 1.00	2.74 ± 0.48	3.92 ± 0.71	2.84 ± 0.55	3.86 ± 0.84	2.64 ± 0.51	3.58 ± 0.71	2.51 ± 0.48	3.53 ± 0.75	2.51 ± 0.55	3.33 ± 0.83	2.37 ± 0.59
**Skeletal muscle radiation attenuation, HU**	49.3 ± 7.40	47.6 ± 7.87	0.001	57.7 ± 6.37	54.9 ± 11.2	53.3 ± 6.84	52.1 ± 6.86	50.6 ± 6.79	50.6 ± 6.23	48.7 ± 6.92	47.6 ± 7.48	46.3 ± 6.83	44.6 ± 8.46	45.5 ± 7.91	42.2 ± 5.22

Values of variables are given as mean ± standard deviation.

Skeletal Muscle Area = total muscle surface area. Total muscle surface area = total abdominal wall muscle surface area + right m. iliopsoas muscle surface area + left m. iliopsoas muscle surface area.

Skeletal Muscle Index = total muscle surface area corrected for height.

Statistical significance is displayed for male vs female.

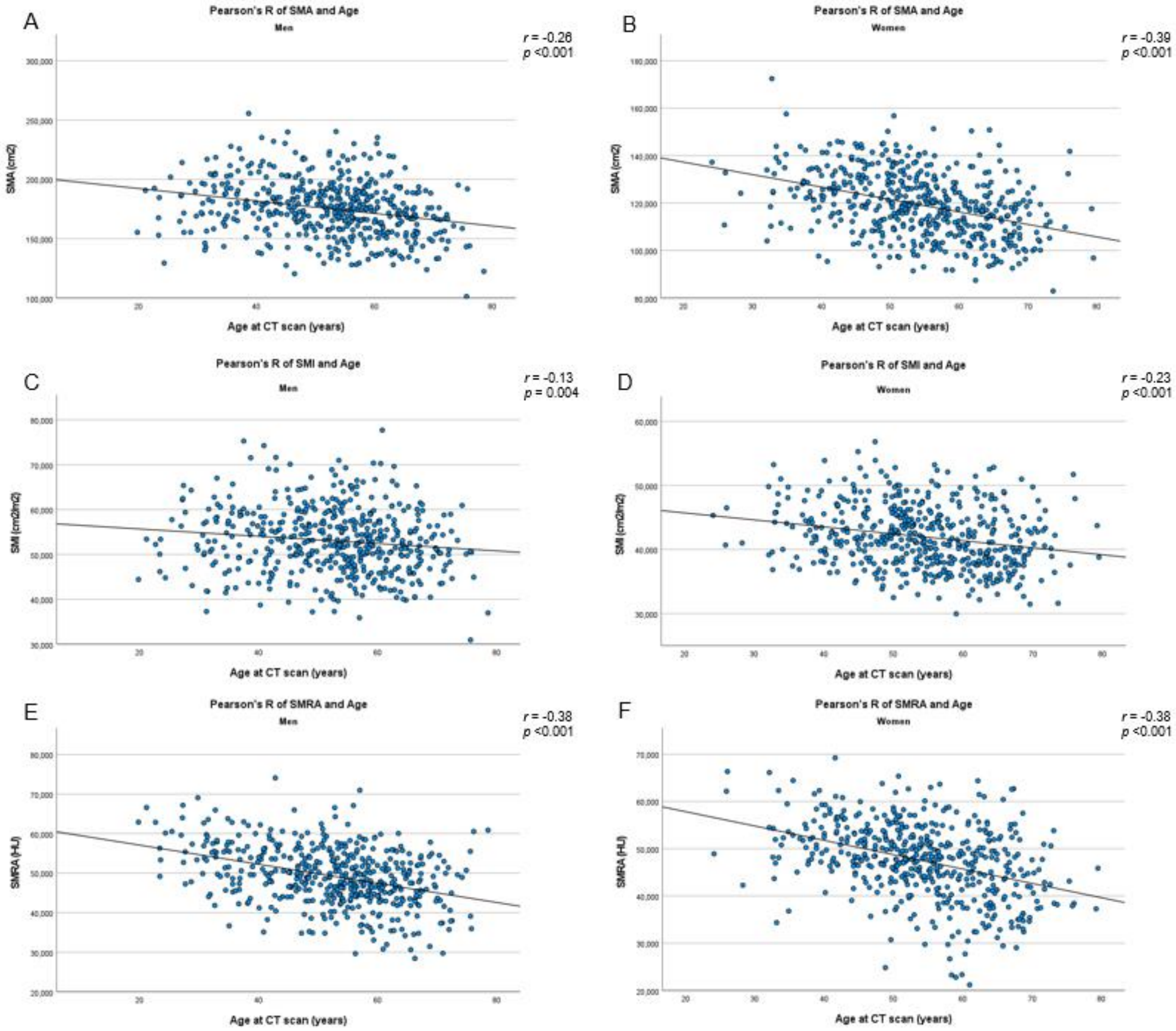

Figure S1. Pearson's correlation SMA, SMI, and SMRA with age at CT scan for men and women. (**A**) Pearson's correlation of skeletal muscle area (SMA) and age at CT scan for men; (**B**) Pearson's correlation of SMA and age at CT scan for women; (**C**) Pearson's correlation of skeletal muscle index (SMI) and age at CT scan for men; (**D**) Pearson's correlation of SMI and age at CT scan for women; (**E**) Pearson's correlation of skeletal muscle radiation attenuation (SMRA) and age at CT scan for men; (**F**) Pearson's correlation of SMRA and age at CT scan for women. r : Pearson's r

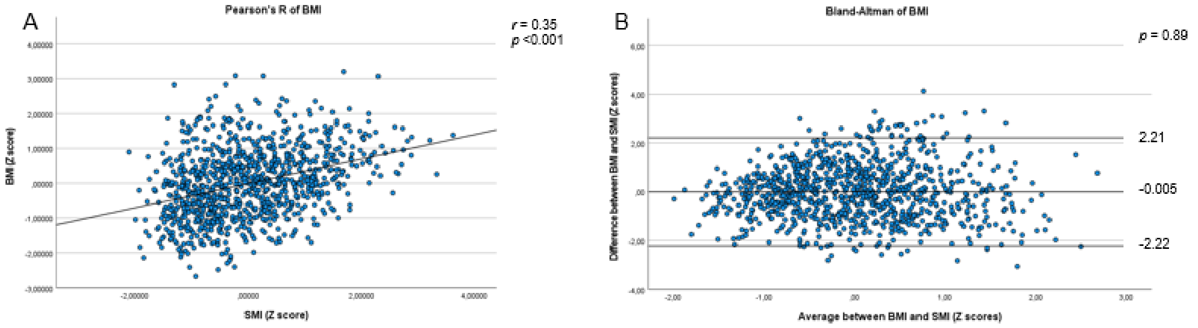

Figure S2. Pearson's correlation and Bland-Altman plots of BMI and SMI. (**A**) Pearson’s correlation of body mass index (BMI) and skeletal muscle index (SMI); (**B**) Bland-Altman plot of BMI and SMI. The middle horizontal line in the Bland-Altman plot shows the mean of the differences (=bias) between the two methods, and the outer two horizontal lines show the upper and lower 95% limits of agreement (= bias ± 1.96 × SD). r : Pearson’s r
